# A new method of rock type identification based on transformer by utilizing acoustic emission

**DOI:** 10.1371/journal.pone.0309165

**Published:** 2024-08-27

**Authors:** Tingting Wang, Yifan Qin, Ranjith P. G., Wanchun Zhao, Jingyi Jiang, Huayi Xu, Xuetong Du

**Affiliations:** 1 School of Electrical Engineering & Information, Northeast Petroleum University, Daqing, China; 2 Heilongjiang Provincial Key Laboratory of Networking and Intelligent Control, Northeast Petroleum University, Daqing, China; 3 Deep Earth Energy Laboratory, Department of Civil Engineering, Monash University, Melbourne, Victoria, Australia; 4 National Key Laboratory of Continental Shale Oil, Northeast Petroleum University, Daqing, Heilongjiang, China; 5 Key Laboratory of continental shale hydrocarbon accumulation and efficient development, Ministry of Education, Daqing, China; Instituto Tecnologico de Aeronautica, BRAZIL

## Abstract

The characterization and analysis of rock types based on acoustic emission (AE) signals have long been focal points in earth science research. However, traditional analysis methods struggle to handle the influx of big data. While signal processing methods combined with deep learning have found widespread use in various process analyses and state identification, effective feature extraction using progressive fusion technology still faces challenges in the field of intelligent rock type identification. To address this issue, our study proposes a novel framework for rock type identification based on AE and introduces a new signal identification model called 3CTNet. This model integrates convolutional neural networks (CNNs) and Transformer encoder, intelligently identifying AE of different rock fractures by establishing dependencies between adjacent positions within the data and gradually extracting advanced features. Furthermore, we experimentally compare five oversampling methods, ultimately selecting the adaptive synthetic sampling method (ADASYN) to balance the dataset and enhance the model’s robustness and generalization ability. Comparison of the internal structure of our model with a series of time series processing models demonstrates the effectiveness of the proposed model structure. Experimental results showcase the high identification accuracy of the intelligent rock type identification model based on 3CTNet, with an overall identification accuracy reaching 98.780%. Our proposed method lays a solid foundation for the efficient and accurate identification of formation rock types in geological exploration and oil and gas development endeavors.

## Introduction

Rock type identification has always been an important topic of research in the fields of geology and resource extraction. Accurate identification of rock information can provide critical data support for geologic analysis, resource assessment, and extraction strategies [1–3], which can not only help to predict subsurface structure [4] and resource distribution [5], but also improve the efficiency of exploration and development, and reduce the risk and cost. Application techniques related to rock type identification include resource assessment, stratigraphic analysis, drilling strategy development [6–8], and reservoir characterization [9], mining technology selection and environmental protection etcetera [10–12]. Therefore, accurately identifying and analyzing the rock structural composition is crucial to effectively develop oil and gas strategies that optimize resource production.

There are many data tools for rock structural analysis, including traditional physical measurements and image-based processing methods, such as electron microscope scanning technology [13], computed tomography (CT) scanner [14], infrared remote sensing [15], numerical simulation [16], electrical impedance [17], rock images [18] and acoustic emission signals (AE) [19] etcetera. Researchers have conducted a series of studies combining various data tools. Shehata, Amer A et al. utilized an artificial neural network (ANN) in combination with core and borehole image data, logging data to identify five rock phases, and clarified the specific parameters that contribute to rock phase identification [20]. Liu, Haiqiang et al. propose a novel hybrid attention semantic segmentation (HASS) network for semantic segmentation of martian terrain rocks for Mars rover route planning and autonomous navigation [21]. These methods of using images to identify rocks may have limitations for structures deep in the earth. As AE measurement instruments become increasingly sophisticated, scholars begun to use AE to explore areas that are visually unreachable [22, 23]. AE refers to the generation of transient elastic waves due to the rapid release of localized energy in a material or structure. For instance, Lee, Choi. et al. proposed an AE processing method based on the Hilbert Huang transform (HHT), and the processed AE can be combined with signal parameters (AESP) to jointly identify rock fracture deformation stages [24]. Zhang et al. utilized short-time Fourier transform (STFT) to analyze the acoustic emission signal and obtain the peak frequency of the signal and used the fuzzy C-means method to classify the four types of acoustic emission signals [25]. These primitive AE processing methods are in urgent need of scientific and technological innovation at the present time, its susceptibility to noise interference, limited processing samples, low accuracy, slow speed, high operating costs and other shortcomings make it difficult to sustain the development. Rock structure identification based on traditional acoustic emission signal analysis methods still dominates, but these methods are gradually becoming auxiliary and are clearly unable to meet the growing engineering demand for high efficiency and high precision. At present, various engineering fields are facing the massive influx of sensor data from job platforms, which is increasing the demand for intelligent signal processing [26, 27]. Certain fundamental artificial intelligence algorithms are steadily gaining popularity, including support vector machines (SVMs) [28], convolutional neural networks (CNNs) [29], and generating adversarial networks (GANs) [30] and so on. The application of intelligent methods greatly improves the recognition efficiency and accuracy while also providing time and labor savings. Such as Byun, H. et al. proposed a method that employs a deep CNN to segment rock crack images, achieving effective recognition by effectively distinguishing cracks from various interference features [31]. However, AE is an unstructured continuous time sequence [32], rock fracture identification requires the determination of more parameters than just images, which result in multimodal data [33]. The recognition of rock activity processes based on AE signals entails analyzing the intricate features of material movement.

Summarize the changes from traditional methods to intelligent methods mentioned above, there are three challenges that require attention: (1) The risk of overfitting when dealing with strongly noisy data. (2) The problem of recognition accuracy, the robustness of the model. (3) Experimental uncertainty leads to unbalanced sample sizes for different categories and scarce labeling data. Given these considerations, we propose a novel method for recognizing rock fracturing acoustic emission signals using a combination of CNNs. Currently, there have been many researches around transformer, but most of them are focused on processing machine language [34, 35] or visual images [36, 37], and there is no method to apply CNNs combined with transformer encoder to rock fracturing AE recognition.

To address these challenges and improve the identification of rock fracture types, this study introduces a computational model called 3CTNet. To tackle the overfitting issues and sample imbalance problems associated with noisy data, the raw data are first wavelet denoised [27]. Solving the problem of imbalanced samples, five data oversampling methods were compared, including random process sampling [29], ADASYN [30], synthetic minority oversampling technique (SMOTE) [31], SVM-SMOTE and Kmeans-SMOTE [32]. Then, the AE are processed using the ADASYN oversampling for the first time, enhancing both the internal features of the data and the distinguishing features between different classes. To enhance the robustness of the model, we conbined CNNs with a transformer encoder and reconstructed the position encoding module. This combination allows the model to extract local features and capture global connections within the data. Additionally, the model employs Dropout, which randomly drops some neurons during the training process to prevent overfitting. For the validation of the new model, we carried out ablation experiments within the model and comparison experiments between the models, and quantitatively analyzed the effects of noise changes and data sample size changes on the robustness of the model, and finally concluded that the model’s recognition accuracy reaches 98.78%, and has good robustness to changes in noise and sample size.

In the following article, the proposed model describes in detail the CNNs part and transformer encoder part of the model as well as the overall architecture and detailed parameters. Method describes the data processing and the rock mechanics analysis based on the AE. Experiments describes the overall implementation process of the architecture, the model comparison experiments and model evaluation indexes. Results and discussion describes the review of the experiments and the analysis and discussion of the results, and Conlusion is the summary of the overall study.

## Proposed model

This section introduces the relevant modules proposed for the AE processing model. The model comprises three sequential convolution modules and a transformer module, leveraging fracturing AE signals for rock type identification. In this study, the proposed model separates the position encoding module of the transformer encoder from the multihead attention module. Initially, position encoding is applied to the original input data, mapping the discrete data representation into a continuous vector space. Subsequently, convolutional feature extraction is conducted on the output information pertaining to the mark’s relative position. These extracted high-level features are then input into the multihead attention layer for rock type classification. This model, termed 3CTNet, is introduced as part of our contributions.

### Feature extraction by CNN

**Fig 1:**
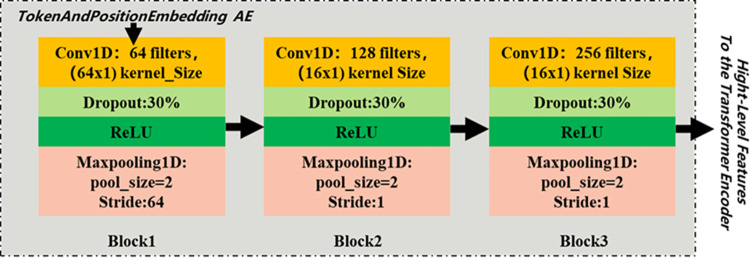
Convolutional operation of AE signals.

To extract high-level representations from AE signals, a CNN module comprising three convolutional layers is utilized for low-level feature extraction. This module consists of three CNNs connected sequentially. Following the addition of positional encoding to each time point record of the original AE signal, discrete time series are transformed into dense embedding vector representations. These representations are then fed into a three-layer concatenated CNNs with filters (64) and kernel_size (64x1) with a stride of 64, filters (128) and kernel_size (16x1) with a stride of 1, and filters (16) and kernel_size (16x1) with a stride of 1. Additionally, a dropout rate of 30% is applied to each layer to mitigate overfitting, and the rectified linear unit (ReLU) [38] activation function is used, defined as *y*_*i*_ = max(0,*x*_*i*_). The softmax output of the classifier calculation is $\begin{array}{c} y_{j}=\frac{e^{x_{j}}}{\sum_{i^{\prime }=1}^{n}e^{x_{i}}} \end{array}$, where n denotes the number of inputs to the neuron. Finally, maximum pooling (MaxPooling) is applied to reduce the feature size, which aids in alleviating model overfitting. The convolutional layer’s operation on the input signal is illustrated in (Fig 1).

### Multihead attention encoder

The utilization of convolutional layer-based transformers is inspired by the success of transformers and their variants in audio signal processing applications [39, 40]. Given the similarity between audio signals and AE signals, the transformer is applied to AE signal recognition. However, transformers typically exhibit high computational complexity. When dealing with time-series data at the basic unit level, significant computing resources are consumed. To address this challenge, a CNN is employed to extract detailed features and preprocess the data before inputting it into the transformer. This approach ensures optimal utilization of the transformer’s advanced computational capabilities.

**Fig 2:**
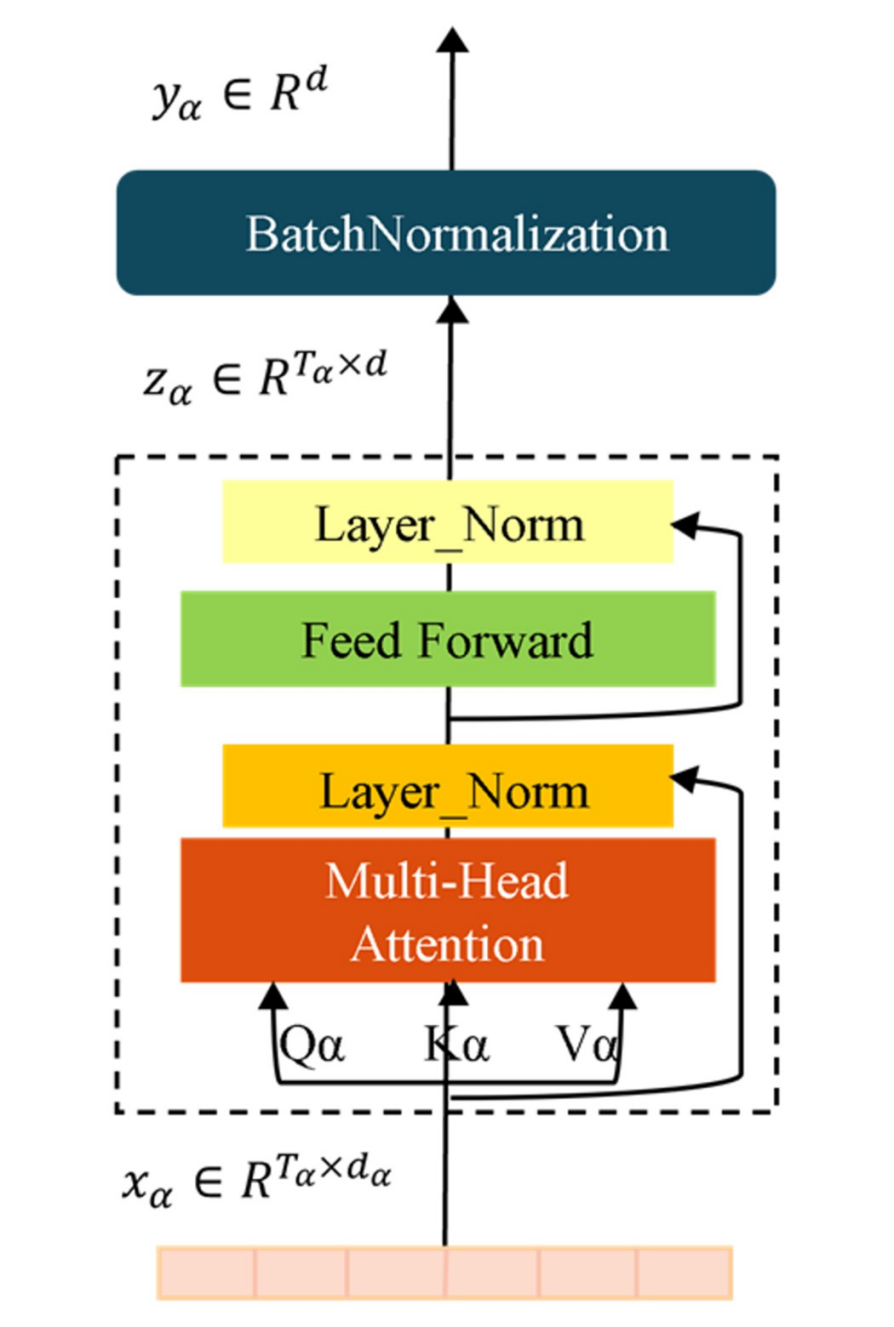
Single mode recognition transformer encoder architecture.

After traversing the convolutional layer, the output *x*_*α*_ size is 2x256. The processed input then undergoes further refinement through the multihead attention layer, followed by weight adjustment via layer normalization and residual connections. Additionally, a feedforward neural network is introduced between the multihead attention layer and the normalization layer. In this configuration, two fully connected layers are utilized to nonlinearly transform the output from the attention module. The constructed encoder architecture is illustrated in (Fig 2).

For multihead attention modules, linearly project the query $Q\in R^{T_{Q}\times d_{Q}}$, key $K\in R^{T_{K}\times d_{K}}$ and value $V\in R^{T_{V}\times d_{V}}$ m times with different linear projections. Here, m is the number of heads:

$$\hat{Q}=Concat(QW_{1}^{Q},\ldots ,QW_{i}^{Q},\ldots ,QW_{m}^{Q})$$ (1)

$$\hat{K}=Contat(KW_{1}^{K},\ldots ,KW_{i}^{K},\ldots ,KW_{m}^{K})$$ (2)

$$\hat{V}=Contat(VW_{1}^{V},\ldots ,VW_{i}^{V},\ldots ,VW_{m}^{V})$$ (3)

where $W_{i}^{Q}\in R^{d_{Q}\times d_{m}},W_{i}^{K}\in R^{d_{K}\times d_{m}}\;\mathrm{and}\;W_{i}^{V}\in R^{d_{V}\times d_{m}}$ are trainable parameters. Here, assuming *d*_*m*_ as the output feature dimension.

On each of these projected versions of queries $QW_{i}^{Q}\in R^{T_{Q}\times d_{m}}$, keys $KW_{i}^{K}\in R^{T_{K}\times d_{m}}$ and values $VW_{i}^{V}\in R^{T_{V}\times d_{m}}$ Next, the dot-product attention is performed in parallel, and the outputs of the attention functions, denoted as *head*_*i*_, *i*∈[1,*m*], are concatenated to obtain the final values *H*_*head*_, which are computed as follows:

$$head_{i}=softmax((QW_{i}^{Q})(KW_{i}^{K}{)}^{T})(VW_{i}^{V})$$ (4)

If the original input signal X is segmented into n segments, each segment undergoes linear transformations separately. Subsequently, the aforementioned steps are executed independently for each segment. Finally, the transformed segments are concatenated to form a complete sequence with the same dimensionality. This process embodies the execution of the multihead attention mechanism:

$$H_{head}=Contat(head_{1},\ldots ,head_{m})$$ (5)

where $head_{i}\in R^{T_{Q}\times d_{m}}\;\mathrm{and}\;H_{head}\in R^{T_{Q}\times md_{m}}.$ Therefore, the sequence length of the outputs Hhead is equal to the sequence length of the query Q.

### Model structure

For the data $Input=X\in R^{T\times d}$ (T is the sequence length, d is the embedding dimension), positional embedding was used to add information to each position of the sequence so that the model could capture the sequential relationship in the sequence, expressed as:

$$\begin{array}{l} Pos\_emb(pos,2i)=sin(\frac{pos}{10000^{2i/d}})\\ Pos\_emb(pos,2i+1)=cos(\frac{pos}{10000^{2i/d}}) \end{array}$$ (6)

where pos was employed to denote the position within the input sequence, with a range of [1, T], and i represented the index of the embedded dimension, with a range of [0, d]. This process involved generating various periodic patterns using sine and cosine functions, and subsequently combining these patterns to generate a code corresponding to a specific position within the input sequence. Subsequently, the position code was incorporated into the input sequence to embed positional information. This process is mathematically represented as follows:

$$Output=sum(Input,{Pos}_{emb})$$ (7)

where Input is the original input matrix and Pos_emb is the positional embedding. After that, *Output* = *X*′∈ℝ^*T*×*d*^. When the data reached the CNN blocks, convolve *X*′ with kernel k in width and height, used ReLU to activate the neurons to obtain the output y:

$$y=X^{\prime }⊗k$$ (8)

Regularize the output y and randomly discard 30% of the neurons, which is expressed as:

$$y^{\prime }=\frac{y*E}{1-p}$$ (9)

where E is a binary mask matrix with the same shape as the input y, and p is the discard probability. Then, these features were passed to the max-pooling layer, the most popular max-pooling operation that divides the input into nonoverlapping parts and obtains the highest gain from each relevant region. The proposed model went through 3 described blocks and finally reached the encoder. Assuming that the arrival sequence is *Y*∈ℝ^*T*×*d*^, the encoding process is expressed as:

$$A=MultiheadAttention(Y,Y){\in R}^{T\times d}$$ (10)

$$B=Dropout(A){\in R}^{T\times d}$$ (11)

$$C=Layer\_Norm(Y,B)\in R^{T\times d}$$ (12)

$$D=FFN(C)\in R^{T\times d}$$ (13)

$$E=Dropout(D)\in R^{T\times d}$$ (14)

$$Output=Layer\_Norm(C,D)\in R^{T\times d}$$ (15)

where FFN is a feedforward neural network and obtains *Output* = *Z*∈ℝ^*T*×*d*^ after encoding. Then, batch normalization was performed on Z, followed by global average pooling to reduce the number of parameters:

$$Z=\frac{1}{T\times d}\sum_{j=1}^{T}\sum_{k=1}^{d}Z_{ijk}\in R^{T\times d}$$ (16)

*Z*_*ijk*_ represents the i-th channel, j-th row, and k-th column element of the input feature matrix. Z represents the i-th feature channel of the output and was sent to two fully connected layers for classification. Then, softmax was used for activation:

$$softmax{\left(Z\right)}_{i}=\frac{e^{Z_{i}}}{\sum_{j=1}^{C}e^{Z_{j}}}$$ (17)

**Fig 3:**
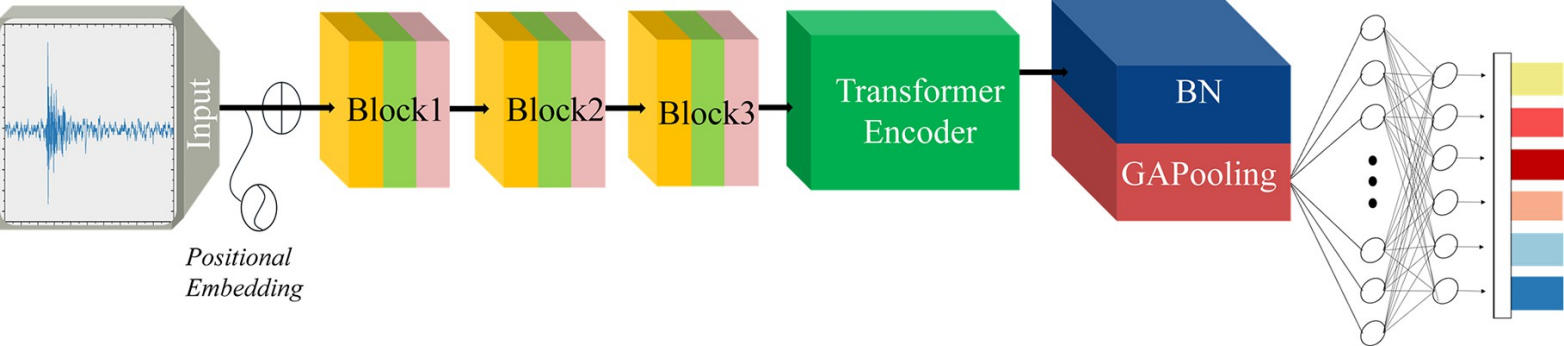
3CTNet for processing AE signals.

*softmax*(*Z*)_*i*_ represents the probability of the i-th category, and C represents the number of categories. In this manner, after fully connected layers and softmax activation, the model output can be expressed as a probability distribution for each category. The detailed model architecture is illustrated in (Fig 3).

The number of layers, convolution kernel size, pooling kernel size, and stride were fine-tuned in the model through iterative trials. The specific parameters of the model are presented in Table 1.

**Table 1:** Model architecture and parameters.

Layes	Types	Activation funciton	Output Shapes	Kernel Size	No.of Filters	Stride	Padding	No.of Trainable Parameters	Schema
**0**	Input	-	1024x1	-	-	-	-	0	-
**1**	Token_and_position_embedding	-	1024x1	-	-	-	-	1024	-
**2**	Conv1D	ReLU	16x64	64x1	64	64	same	4160	Dropout(30%)
**3**	MaxPooling1D	-	8x64	2x1	64	-	-	0	-
**4**	Conv1D	ReLU	8x128	16x1	128	1	same	131200	Dropout(30%)
**5**	MaxPooling1D	-	4x128	2x1	128	-	-	0	
**6**	Conv1D	ReLU	4x128	16x1	128	1	same	524544	Dropout(30%)
**7**	MaxPooling1D	-	2x256	2x1	256	-	-	0	-
**8**	TransformerBlock	-	2x256	-	-	-	-	26009	-
**9**	Batchnormalization	-	2x256	-	-	-	-	1024	-
**10**	GlobalAveragePooling1D	-	256	-	-	-	-	0	Dropout(30%)
**11**	Dense	Softmax	60	-	-	-	-	15420	Dropout(30%)
**12**	Dense	Softmax	6	-	-	-	-	366	-
**Params**								Total params:703747	Trainable params:703235

## Method

### Data preprocessing

#### Datasets and statistical characteristics

**Fig 4:**
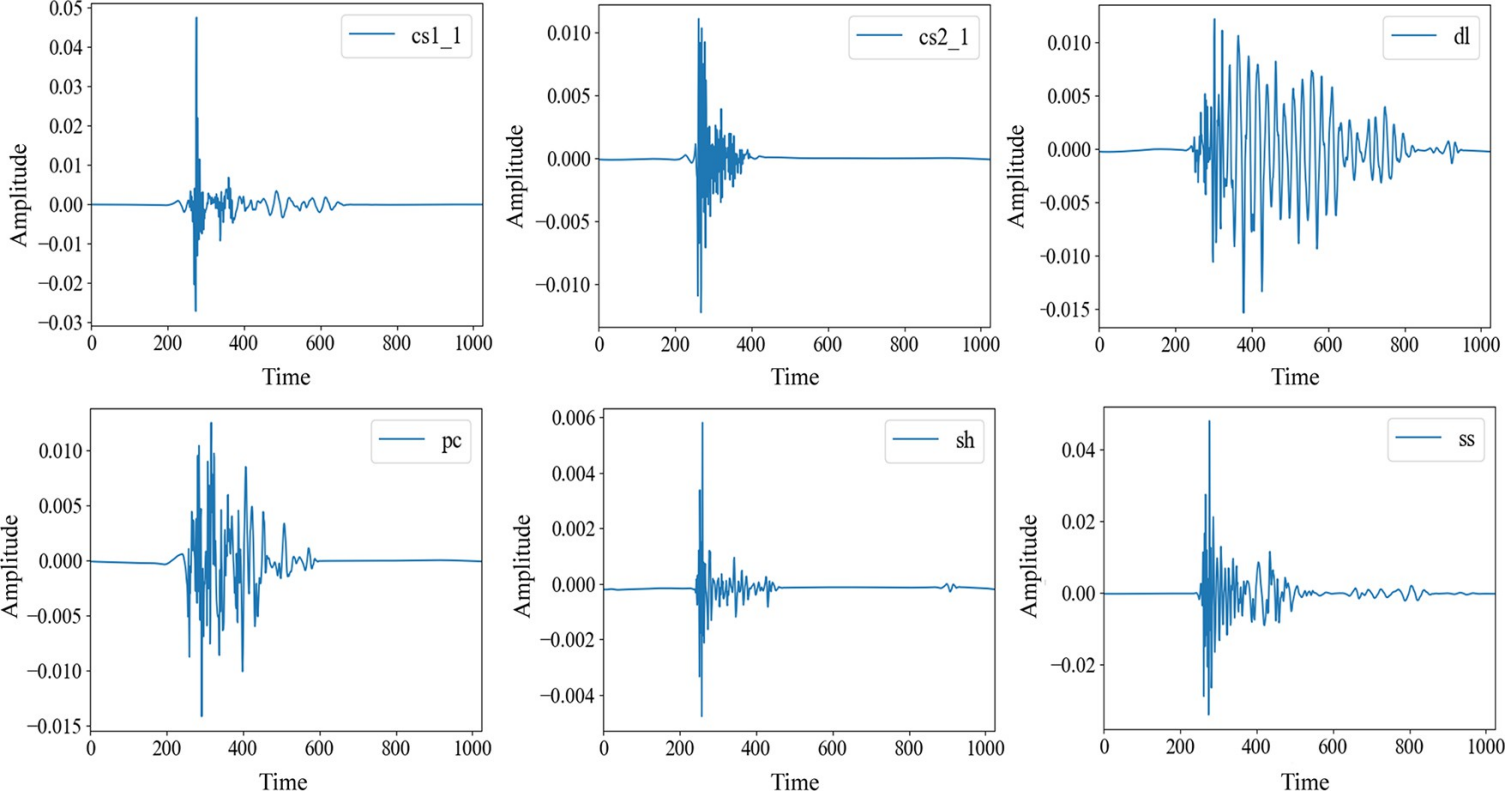
AE representation of Brazilian splitting of six types of rocks.

**Fig 5:**
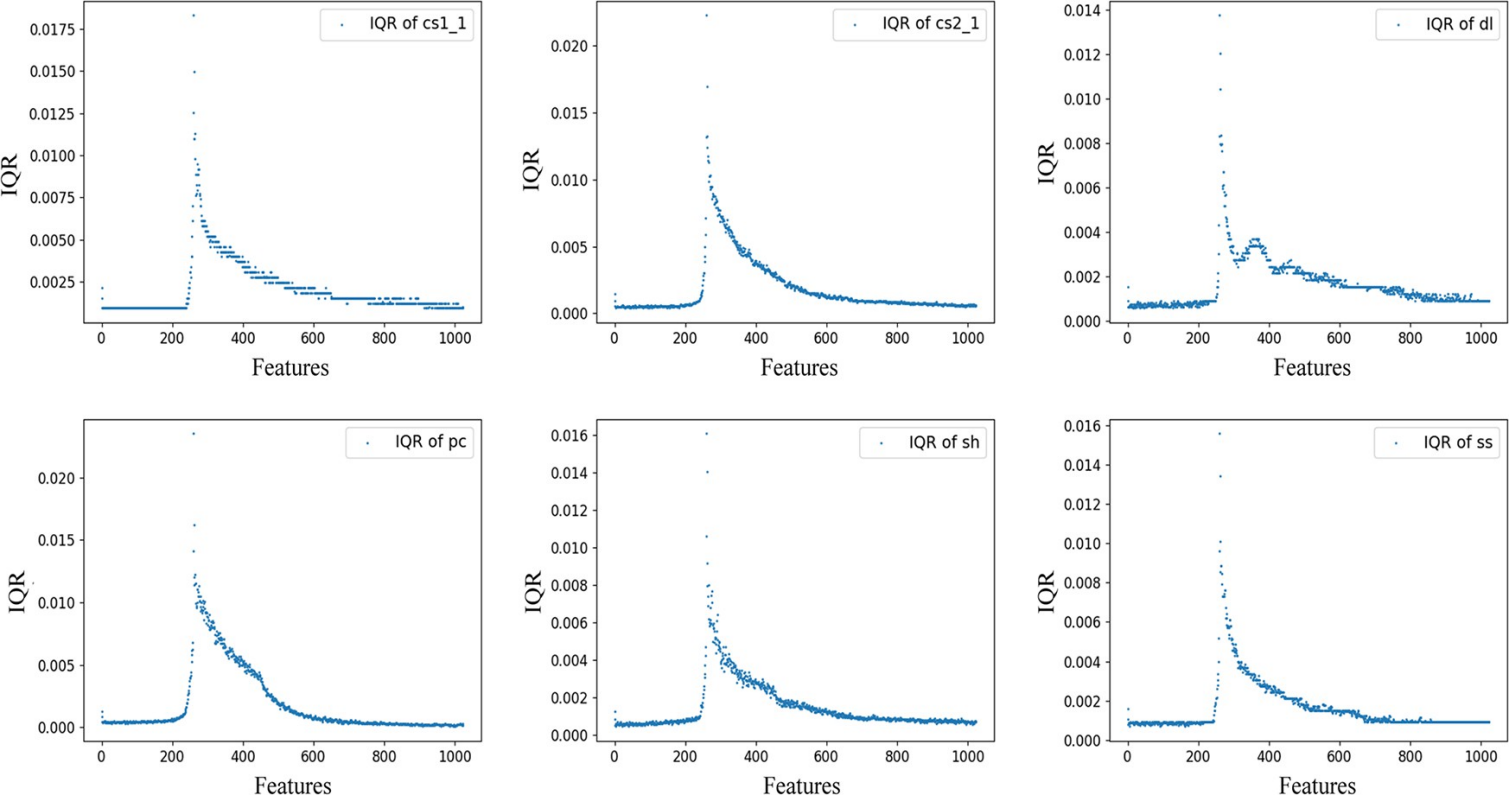
Representing the distribution shape of data through IQR.

**Fig 6:**
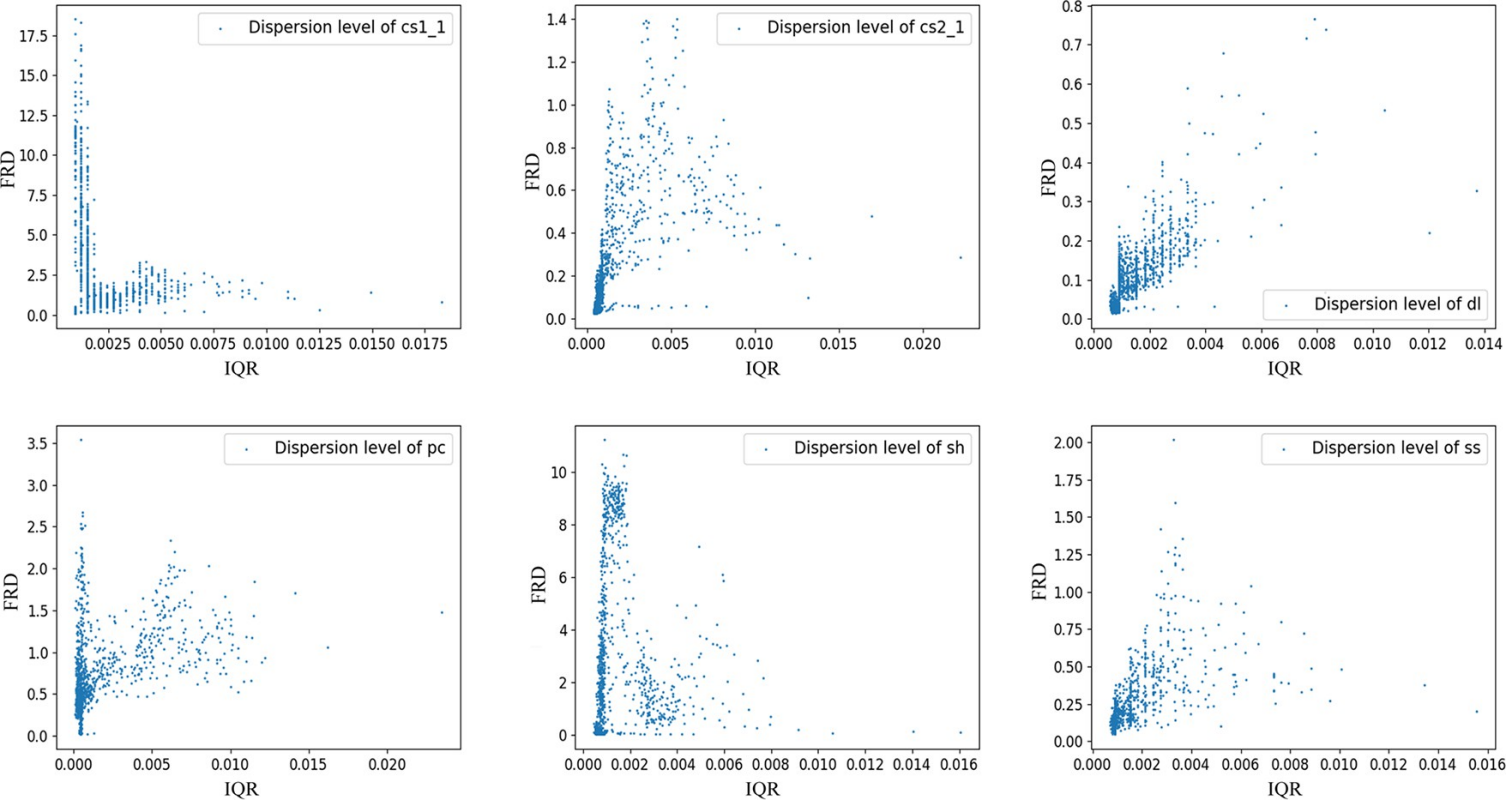
Degree of dispersion of data.

The AE data processed for the experiments were obtained from the Brazilian splitting tests conducted by Song et al. [41] on six sets of rock samples prepared in the laboratory. These specimens comprised three types of natural rocks with different mineral compositions and moduli of elasticity: dolomite (mainly composed of dolomite and calcite) (dl), sandstone (mainly composed of quartz and feldspar) (ss) and shale (mainly composed of quartz, kaolinite and pyrite) (sh). Three types of artificial rocks with different particle contents, pure cement (pc), cement and sand (1:1) (cs:1) and cement and sand (2:1) (cs2:1), formed rock-like concrete, and data visualization is shown in (Fig 4). Subsequently, the interquartile range (IQR) was employed to analyze the statistical characteristics of the dataset and observe its distribution shape, as depicted in (Fig 5). The IQR was compared with the full range (difference between maximum and minimum values) (FRD) of the data to glean information regarding the degree of data dispersion, as illustrated in (Fig 6). It is evident that the overall characteristics of the dataset exhibit a small IQR but a large FRD, indicating that the data are concentrated in the middle area with notable outliers or abnormal values, considerable overall variation, and uneven distribution.

The tests were conducted by treating each of the six specimens as a disk with a thickness-to-diameter ratio of 1:2. To collect the AE data released by each specimen after Brazilian splitting, eight AE transducers were placed on the surface of each disk specimen, three on the front and back sides and two on the sides. Following the operating principles of the sensors and the rock fracture mechanism, AE data were collected from the fracture of the six rock specimens. Each dataset comprises signal amplitudes measured at 1024 consecutive time points.

#### Wavelet threshold denoising

To mitigate noise in the original AE signal, wavelet threshold denoising was utilized. This method involves separating the low-frequency and high-frequency signals and then effectively processing the high-frequency signals to isolate noise from the main information, thus preserving important features. The wavelet transform is a well-suited approach for signal and image denoising, characterized by its ability to efficiently capture both temporal and frequency-domain information. The principle of the wavelet transform can be succinctly expressed as follows:

$$F_{\left(a,b\right)}=\int_{-\infty }^{+\infty }f(t)\Psi^{*}(t;a,b)dt$$ (18)

where the wavelet basis functions are:

$$\Psi^{*}(t;a,b)=\frac{1}{\sqrt{a}}\Psi \left(\frac{t-b}{a}\right)$$ (19)

The study employs the Daubechies 4 (db4) wavelet basis function for the denoising process. The selection of the 4th order wavelet basis function was made after comparing it with other order wavelet functions. It was observed that the db4 function effectively preserves crucial signal information while efficiently filtering out noise. Through the application of this denoising technique, clean AE signal data are obtained, minimizing any potential impact on the accuracy of the model training.

#### ADASYN oversampling

The acquired dataset, influenced by the physical properties of the rock and various factors during testing, is unevenly distributed. This uneven distribution has led to poor detection results for certain classes of samples, thereby affecting the overall recognition accuracy of the model [42]. To address this issue, the dataset was processed by filtering out the more disturbed data from the six sample classes. Subsequently, five data oversampling methods were validated: random process sampling, ADASYN, SMOTE, SVM-SMOTE, and Kmeans-SMOTE. It was found that when the model was trained on the dataset obtained using the ADASYN oversampling method, it achieved better fitting and higher recognition accuracy on a real test dataset. The ADASYN oversampling method accomplished two critical objectives: it mitigated the learning bias introduced by the initial data imbalance and dynamically adjusted the decision boundary to prioritize challenging-to-learn samples [43].

For the ADASYN sampling method, suppose the AE training dataset has m samples: {*x*_*i*_, *y*_*i*_}, (i = 1,2,3,…,m) where *x*_*i*_ is an instance in the n-dimensional feature space X, *y*_*i*_∈*Y* = {1,−1} is the category label associated with *x*_*i*_, and *m*_*s*_ and *m*_*l*_ are defined as the number of minority samples and the number of majority samples, respectively. Then, it follows that *m*_*s*_≤*m*_*l*_ and *m*_*s*_+*m*_*l*_ = *m*. The specific implementation process of the ADASYN algorithm:

1) Calculate the degree of category imbalance:

$$d=m_{s}/m_{l}$$ (20)

where *d*∈[0,1].

2) if *d*<*d*_*th*_ (*d*_*th*_ is a preset threshold for the maximum tolerated degree of class imbalance ratio) then:

(a) Calculate the number of synthetic samples that need to be generated for the minority class:

$$G=(m_{l}-m_{s})\times \beta$$ (21)

where *β*∈[0, 1] is used to specify the expected balance level of the dataset after synthesizing the data. *β* = 1 means a fully balanced dataset is created after the generalization process.

(b) For each sample *x*_*i*_∈*minorityclass*, ind the K nearest neighbors based on Euclidean distances in n-dimensional space and compute the ratio *r*_*i*_, defined as:

$$r_{i}=\frac{\Delta_{i}}{K},i=1,\ldots ,m_{s}$$ (22)

where Δ_*i*_ is the number of samples belonging to the majority class among the *K* nearest-neighbor samples of *x*_*i*_; hence, *r*_*i*_∈[0,1];

(c) Normalize *r*_*i*_ according to ${\hat{r}}_{i}=r_{i}∕\sum_{i=1}^{m_{s}}r_{i}$, so that ${\hat{r}}_{i}$ is a density distribution ($\sum {\hat{r}}_{i}=1$);

(d) Calculate the sample size of synthetic data needed for each minority class sample *x*_*i*_:

$$g_{i}=\hat{r_{i}}\times G$$ (23)

where G is the total number of data samples to be synthesized for the minority class defined in Eq (8);

(e) For each minority sample, example *x*_*i*_ generates *g*_*i*_ synthetic sample examples according to the following steps:

Perform a loop from 1 to *g*_*i*_:

(i) Randomly select a minority sample example *x*_*zi*_ from the K nearest neighbor samples of the data *x*_*i*_.

(ii) Generate a synthetic sample example:

$$s_{i}=x_{i}+(x_{zi}-x_{i})\times \lambda$$ (24)

where (*x*_*zi*_−*x*_*i*_) is the difference vector in n−dimensional space, *λ*∈[0,1], and finally ends the loop [43].

Here, for a better understanding of ADASYN, calculate the degree of imbalance d for each category of imbalance first, then elect the category with the highest degree of imbalance from all imbalanced categories and calculate K nearest samples for each sample using Euclidean distance. Calculate the imbalance ratio for each minority category sample *r*_*i*_ and determine the number of *g*_*i*_ to be synthesized. Create composite samples using random interpolation or other methods between a few category samples and their K-nearest neighbor samples, then merge the synthesized samples with a few category samples from the original dataset to create a new balanced dataset. This process is repeated until all categories of the dataset are balanced.

**Fig 7:**
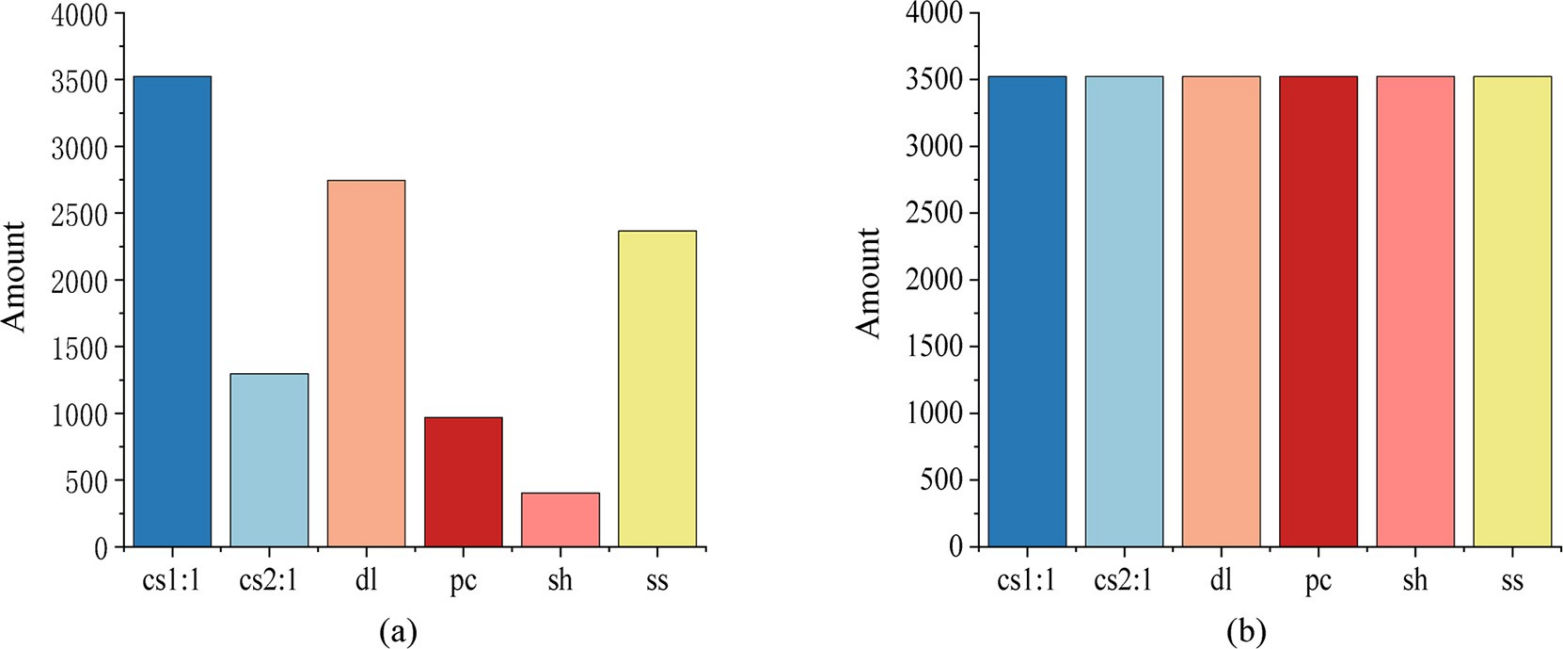
Distribution characteristics before and after data processing. (a) Dataset distribution before oversampling. (b) Dataset distribution after oversampling.

The ADASYN oversampled dataset not only achieves a balanced representation of the data distribution but also forces the learning algorithm to focus on sample classes that are difficult to learn features from. After filtering out data with obvious interference from the original dataset, the ADASYN oversampling method was selected to address the dataset imbalance.This resulted in a dataset with a reasonable sample size and a uniform data distribution, as depicted in (Fig 7). Finally, the total data samples were divided in an 8 : 2 (train : test) ratio for input into the model for training.

### Rock mechanics analysis based on AE

**Fig 8:**
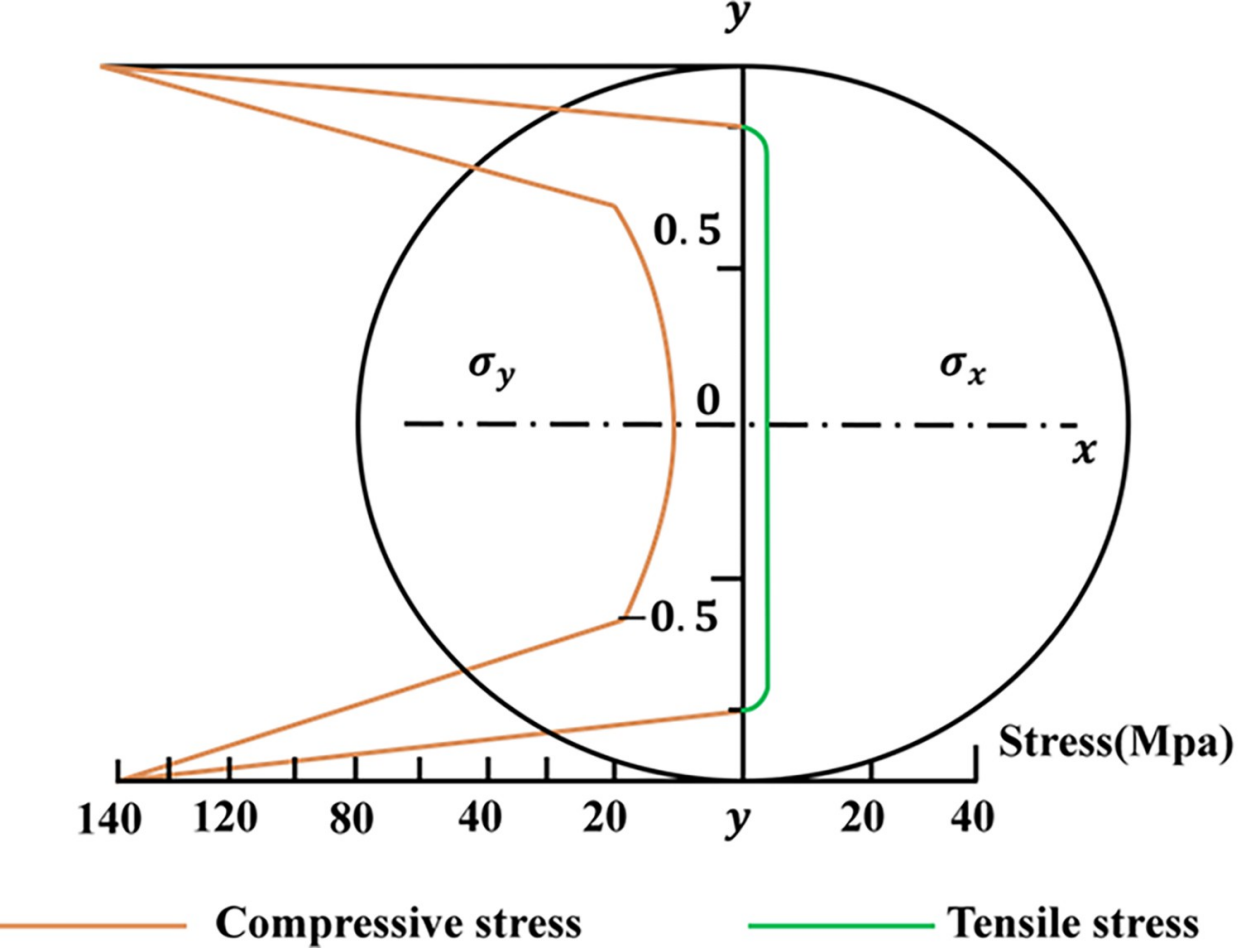
Stress state of a potential fracture under a Brazilian splitting test.

The Brazilian split test induces a tensile stress perpendicular to the vertical diameter that is essentially constant in the area around the center of the specimen [44], The crack stress state under the Brazilian splitting test is shown in (Fig 8). Indirect tensile strength is usually calculated based on the assumption that failure occurs at the point of maximum tensile stress, i.e., the center of the disc. The calculation formula of Brazilian split tensile strength *σ*_*t*_ (MPa) is expressed as:

$$\sigma_{t}=\frac{2P}{\pi Dt}=0.636\frac{P}{Dt}$$ (25)

where P represents the load at failure, D is the diameter of the specimen, and t is the thickness of the specimen measured at the center point [45].

The AE signal used in this study originates from the elastic waves released from multi-scale cracks in rock materials after being subjected to Brazilian cleavage loading. At different stages of rock fracture, the AE signal will exhibit different waveform characteristics. According to existing research and analysis [46, 47], the whole process of rock fracture can be defined as five stages: (1) crack closure stage (2) linear elastic stage (3) crack initiation threshold and stable crack development stage (4) critical energy release and unstable crack development stage (5) rupture and post-peak stage. When a rock receives external force, tiny cracks released at different stages will form inside or on the surface of the rock and release weak acoustic signals. When the stress reaches the critical point of material rupture, the cracks accelerate and expand, thereby generating more AE signals. The quantity and characteristic intensity of these signals can determine whether cracks have occurred. Rock samples with different chemical compositions have different physical properties, so fracturing tests on different samples will result in AE signals with different characteristics. Finally, the rupture modes corresponding to different types of specimens can be inferred from the AE signals, and provide a data basis for feature training of deep learning.

## Experiments

### Workflow

**Fig 9:**
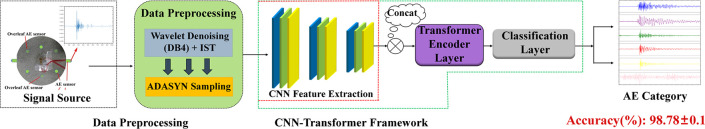
AE signal processing and recognition workflow based on 3CTNet.

This study presents a workflow framework for rock type recognition based on acoustic emission (AE) signals, as depicted in (Fig 9). The framework comprises three main components: data preprocessing, CNN feature extraction, and a transformer encoder. The data preprocessing involves denoising and standardization. Denoising is performed using wavelet thresholding with the db4 wavelet basis function to reduce noise in the original signal. Specifically, the soft thresholding (IST) method is employed, where wavelet coefficients smaller than 3 x *σ* are set to zero, and larger coefficients undergo special treatment, resulting in a smoother denoised signal that effectively filters out subtle noises. Following denoising, standardization is applied using the StandardScaler method. This method scales the data to a normal distribution with a mean of 0 and a standard deviation of 1, ensuring that all sample features are on the same scale. This is advantageous for classification problems requiring distance-based similarity measurements, where the StandardScaler method has demonstrated superior performance.

To address dataset imbalances, the ADASYN oversampling method is employed post-denoising to further balance the data distribution. This prepares the data for deep learning model training and testing. Following continuous fine-tuning, the feature extraction component establishes an optimal structure using a three-layer CNN. Each layer applies the ReLU activation function for nonlinear transformation of input information, which is then passed to the next layer. To enhance model generalization, a dropout layer follows each convolutional layer, selectively deactivating neurons. Additionally, a MaxPooling layer is included to further improve generalization capabilities.

The final architecture connects the output of the CNN feature extraction to a transformer encoder and subsequently to the classification layer. The transformer encoder segments the input sequence, assigning weight information to critical feature regions in each segment to enhance the model’s focus on key features. This structure facilitates efficient recognition by the final classification layer for each type of rock based on AE signals, providing both individual class recognition accuracy and overall model accuracy.

### Model training and comparative experiment

#### Ablation experiment

**Fig 10:**
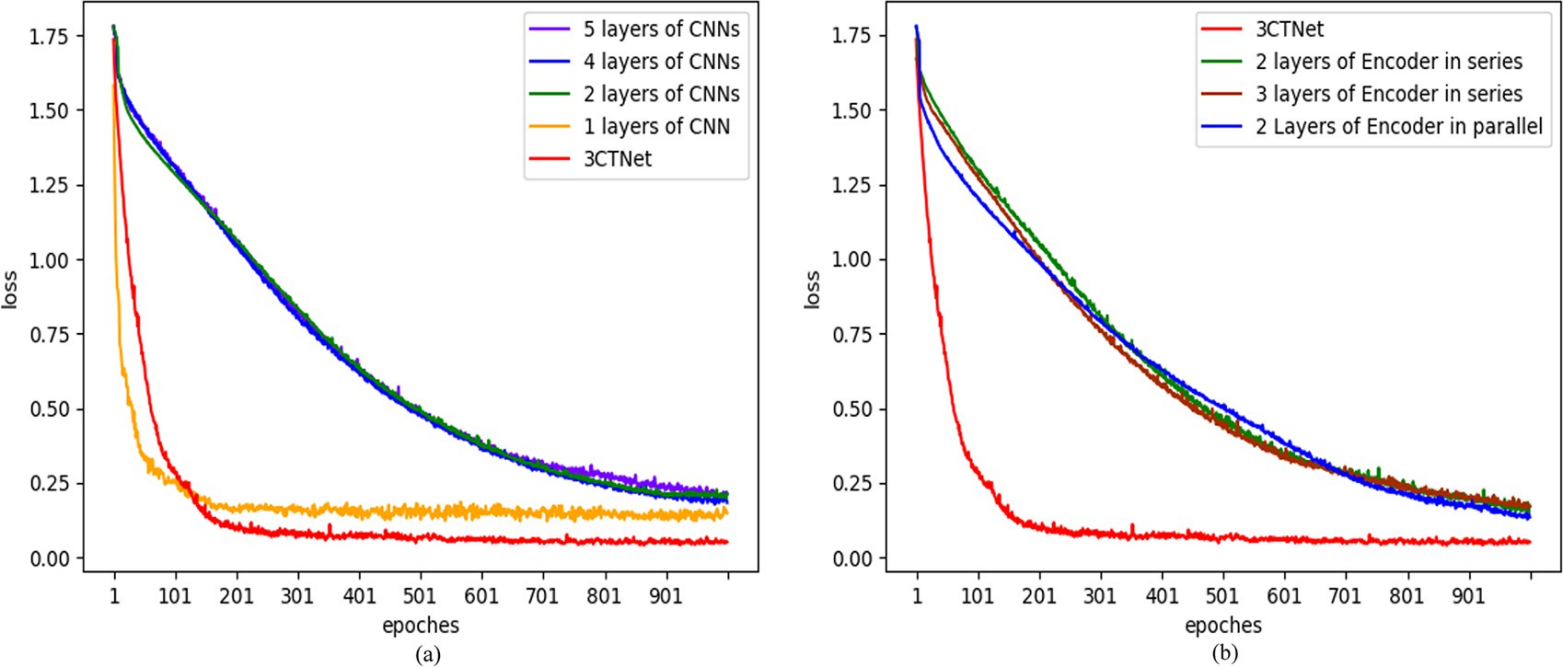
Loss curve in different structures. (a) Convolutional layer comparison. (b) Encoder layer comparison.

The groups of models for which the ablation experiments were performed were first identified based on the structural features of the proposed models as one layers of CNN, two layers of CNNs, four layers of CNNs, six layers of CNNs, two layers of encoder in series, two layers of encoder in parallel, three layers of encoder in series. The hyperparameters of the model were determined by comparison experiments, and the experimental data are shown in Table 2. The denoised data only were input into the above model for recognition comparison respectively, the comparison accuracy of the model is shown in Table 3, and the training loss curve is shown in (Fig 10).

**Table 2:** Hyperparameterization process for different models’ structure.

Model	Accuracy(%)								
	Epoches			Batch size			Learning rate		
	100	500	1000	64	128	256	1.8e-3	1.8e-4	1.8e-5
**1 layers of CNN**	63.882	82.317	87.179	88.462	89.318	86.826	88.196	88.683	89.318
**2 layers of CNNs**	70.380	85.897	88.594	87.931	88.594	86.737	89.125	90.760	88.594
**4 layers of CNNs**	71.618	65.915	87.887	89.302	87.887	86.914	89.169	89.567	87.887
**5 layers of CNNs**	61.671	83.864	87.312	88.373	87.312	86.516	88.373	89.125	87.312
**2 layers of encoder in series**	69.584	81.919	88.019	88.550	88.019	80.990	87.533	89.832	88.019
**2 layers of encoder in parallel**	66.004	79.310	87.179	88.859	87.179	85.853	88.727	89.965	87.179
**3 layers of encoder in series**	61.583	82.095	89.610	82.847	89.610	82.582	89.478	89.610	82.847
**3CTNet**	82.184	89.478	90.186	89.699	90.186	90.053	31.698	90.186	88.638

**Table 3:** Accuracy of different structures of the model.

Self comparison (CNN)	Accuracy(%)	Self comparison (Ecoder)	Accuracy(%)
**1 layers of CNN**	89.07±1.06	**2 layers of encoder in series**	87.93±0.85
**2 layers of CNNs**	86.92±1.32	**2 layers of encoder in parallel**	86.86±1.46
**4 layers of CNNs**	85.78±0.79	**3 layers of encoder in series**	88.56±0.24
**5 layers of CNNs**	86.04±0.72	**3CTNet**	90.15±0.55

From tables and loss curves obtained from the experiments, determined the optimal hyperparameter collocation for learning rate is 1.8e-4, batch size is 128, and number of epochs is 1000. Meanwhile, it is evident that utilizing only a single layer of CNN within the model results in a relatively simple architecture that converges quickly, but does not achieve optimal final convergence. To balance computational resource constraints with model performance, a three-layer CNNs configuration was found to offer the best convergence. By adjusting the structure of the transfomer encoder collocation, it is finally determined that the effect of using one layer of encoder is optimal, so it can be concluded that for the original denoised data, our proposed model 3CTNet has the optimal recognition effect.

#### Data augmentation experiment

The confusion matrix is a valuable tool for visualizing and evaluating the performance of a classification model. It displays the true class labels in the rows and the predicted class labels in the columns, providing four key metrics to assess the model’s predictions against the true classes, as shown in Table 4. True positive (TP) represents the model correctly predicts positive examples as positive examples. True negative (TN) represents the model correctly predicts negative examples as negative examples. False positive (FP) represents the model incorrectly predicts negative examples as positive examples. False negative (FN) represents the model incorrectly predicts positive examples as negative examples.

**Table 4:** Classification confusion matrix.

Confusion Matrix		Predicted Class	
		Predicted Value: Positive(+)	Predicted Value: Negative(-)
**Actual** **Class**	**Actual Value:** **Positive(+)**	True Positive(TP)	False Negative(FN)
	**Actual Value:** **Negative(-)**	False Positive(FP)	True Negative(TN)

These metrics allow for a comprehensive evaluation of the model’s performance in terms of both correct and incorrect predictions.

The accuracy is obtained by dividing the sum of true and true negative values by the sum of positive and negative values in Eq (26), the precision is obtained by dividing the true value by the sum of true and false positive values in Eq (27), and the recall is obtained by dividing the sum of all true values by the sum of all true values and false negative values:

$$accuracy=\frac{TP+TN}{P+N}$$ (26)

$$precision=\frac{TP}{TP+FP}$$ (27)

$$recall=\frac{TP}{TP+FN}$$ (28)

The f1-score evaluation metric, which consists of precision and recall, is a harmonic mean between precision and recall in the evaluation process and can be more useful when dealing with datasets with a large number of categories [48]:

$$f1-score=\frac{2^{*}Precision^{*}Recall}{Precision+Recall}$$ (29)

**Fig 11:**
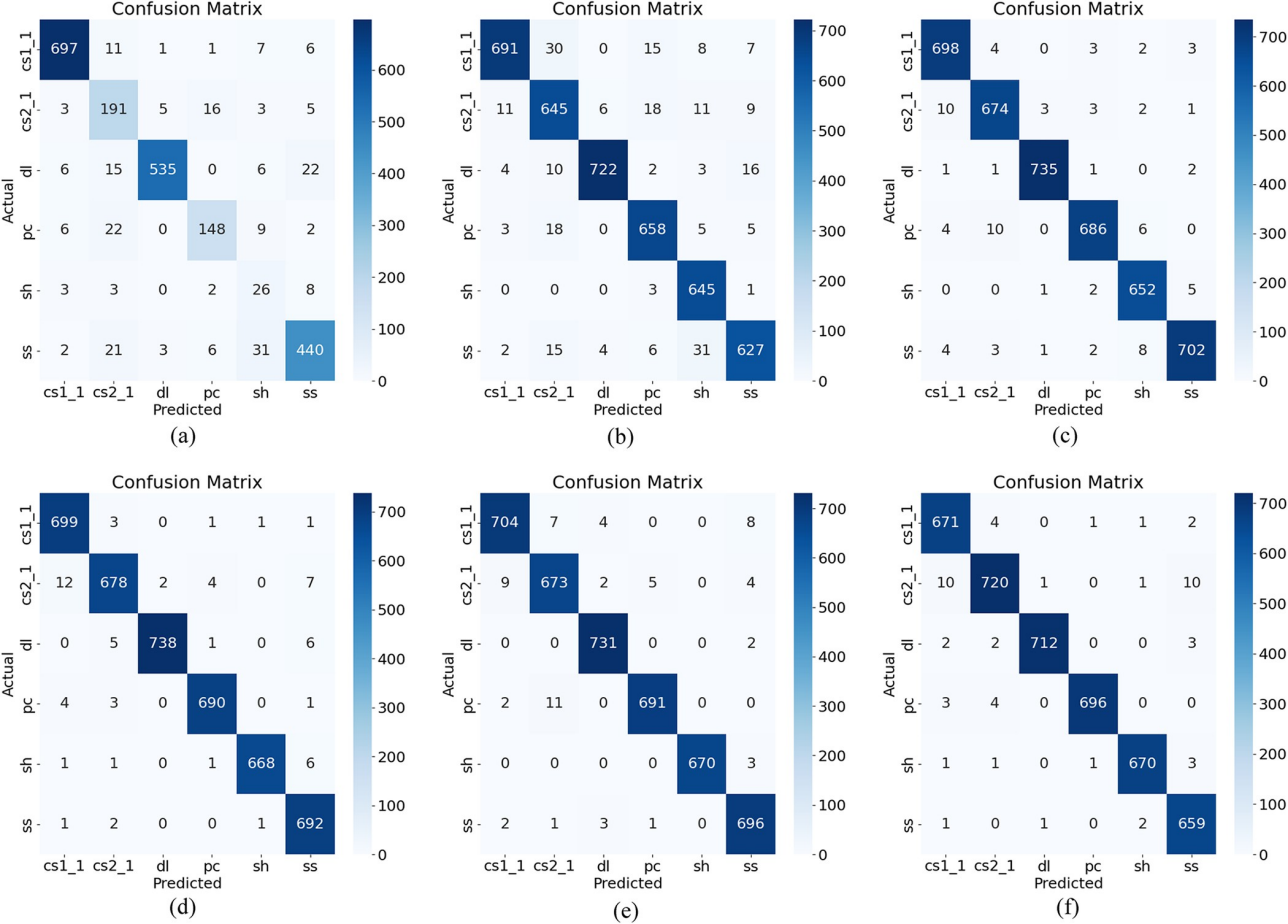
Confusion matrices of 3CTNet training. (a) Original dataset. (b) Kmeans-SMOTE oversampling dataset. (c) SVM-SMOTE Oversampling dataset. (d) SMOTE Oversampling dataset. (e) Random process sampling dataset. (f) ADASYN Oversampling dataset.

The initial recognition accuracy achieved by training the new model with denoised data is 90.15±0.55(%). Subsequently five data enhancement methods including Kmeans-SMOTE, SVM-SMOTE, SMOTE, random process sampling and ADASYN were compared respectively.And our model was used to process six sets of data including the raw data.The experimental comparison confusion matrix is shown in (Fig 11) and the detailed parameters of model and dataset evaluation are shown in Table 5. In the confusion matrix, the diagonal lines indicate the recognition accuracy of the various AE signals, with darker colours indicating higher accuracies.

**Table 5:** Comparison of performance improvement of 3CTNet before and after data oversampling.

**Class**	**cs1:1**	**cs2:1**	**dl**	**pc**	**sh**	**ss**		**Precision**	**Recall**	**F1-Score**	**Support**
**cs1:1**	697	11	1	1	7	6	**cs1:1**	0.97	0.96	0.97	723
**cs2:1**	3	191	5	16	3	5	**cs2:1**	0.73	0.86	0.79	223
**dl**	6	15	535	0	6	22	**dl**	0.98	0.92	0.95	584
**pc**	6	22	0	148	9	2	**pc**	0.86	0.79	0.82	187
**sh**	3	3	0	2	26	8	**sh**	0.32	0.62	0.42	42
**ss**	2	21	3	6	31	440	**ss**	0.91	0.87	0.89	503
**3CTNet with original dataset Accuracy: 90.053%**											
**Class**	**cs1:1**	**cs2:1**	**dl**	**pc**	**sh**	**ss**		**Precision**	**Recall**	**F1-Score**	**Support**
**cs1:1**	691	30	0	15	8	7	**cs1:1**	0.97	0.92	0.95	731
**cs2:1**	11	645	6	18	11	9	**cs2:1**	0.90	0.92	0.91	700
**dl**	4	10	722	2	3	16	**dl**	0.99	0.95	0.97	757
**pc**	3	18	0	658	5	5	**pc**	0.94	0.96	0.95	689
**sh**	0	0	0	3	645	1	**sh**	0.92	0.99	0.95	649
**ss**	2	15	4	6	31	627	**ss**	0.94	0.92	0.93	685
**3CTNet with Kmeans- SMOTE oversampling Accuracy: 94.257%**											
**Class**	**cs1:1**	**cs2:1**	**dl**	**pc**	**sh**	**ss**		**Precision**	**Recall**	**F1-Score**	**Support**
**cs1:1**	698	4	0	3	2	3	**cs1:1**	0.97	0.98	0.98	710
**cs2:1**	10	674	3	3	2	1	**cs2:1**	0.97	0.96	0.96	693
**dl**	1	1	735	1	0	2	**dl**	0.99	0.99	0.99	740
**pc**	4	10	0	686	6	0	**pc**	0.98	0.97	0.98	706
**sh**	0	0	1	2	652	5	**sh**	0.97	0.99	0.98	660
**ss**	4	3	1	2	8	702	**ss**	0.98	0.97	0.98	720
**3CTNet with SVM-SMOTE oversampling Accuracy: 96.801%**											
**Class**	**cs1:1**	**cs2:1**	**dl**	**pc**	**sh**	**ss**		**Precision**	**Recall**	**F1-Score**	**Support**
**cs1:1**	699	3	0	1	1	1	**cs1:1**	0.98	0.98	0.98	705
**cs2:1**	12	678	2	4	0	7	**cs2:1**	0.97	0.97	0.97	703
**dl**	0	5	738	1	0	6	**dl**	1.00	0.99	0.99	750
**pc**	5	3	0	690	0	1	**pc**	0.99	0.98	0.99	699
**sh**	1	1	0	1	668	6	**sh**	0.99	0.99	0.99	677
**ss**	1	2	0	0	1	692	**ss**	0.97	0.98	0.98	696
**3CTNet with SMOTE oversampling Accuracy: 96.203%**											
**Class**	**cs1:1**	**cs2:1**	**dl**	**pc**	**sh**	**ss**		**Precision**	**Recall**	**F1-Score**	**Support**
**cs1:1**	698	4	0	3	2	3	**cs1:1**	0.97	0.99	0.98	710
**cs2:1**	10	674	3	3	2	1	**cs2:1**	0.98	0.97	0.97	693
**dl**	1	1	735	1	0	2	**dl**	0.99	0.99	0.99	740
**pc**	4	10	0	686	6	0	**pc**	0.99	0.98	0.99	697
**sh**	0	0	1	2	652	5	**sh**	1.00	1.00	1.00	660
**ss**	4	3	1	2	8	702	**ss**	0.98	0.99	0.99	720
**3CTNet with random process sampling Accuracy: 97.652%**											
**Class**	**cs1:1**	**cs2:1**	**dl**	**pc**	**sh**	**ss**		**Precision**	**Recall**	**F1-Score**	**Support**
**cs1:1**	699	3	0	1	1	1	**cs1:1**	0.98	0.98	0.98	705
**cs2:1**	12	678	2	4	0	7	**cs2:1**	0.98	0.97	0.97	703
**dl**	0	5	738	1	0	6	**dl**	1.00	1.00	1.00	750
**pc**	4	3	0	695	0	1	**pc**	1.00	0.99	0.99	703
**sh**	1	1	0	1	668	6	**sh**	1.00	0.99	1.00	677
**ss**	1	2	0	0	1	692	**ss**	0.98	1.00	0.99	669
**3CTNet with ADASYN oversampling Accuracy: 98.876%**											

The model recognition accuracy increased to 94.257%, 96.801%, 96.203%, 97.652% and 98.876% respectively for the data processed by the five data enhancement methods. Among those methods, ADASYN demonstrated the highest performance, achieving a recognition accuracy about 98.876%. Based on the comparison of the results, the ADASYN oversampling method was ultimately selected as the most suitable approach for balancing the rock AE dataset, as it resulted in the highest recognition accuracy.

Table 5 contains the parameters of accuracy, precision, recall, f1-score, etc. Finally, as we can see, after ADASYN processing of the dataset and inputting the model for recognition, the precision, recall, and f1-score of four specimens of dl, pc, sh ss reached 100%, and the evaluation indicators of other two samples reached almost 100%. By analyzing these evaluation indicators, we can conclude that the constructed model of ADASYN data process exhibits superior recognition performance.

#### Model performance and evaluation

**Fig 12:**
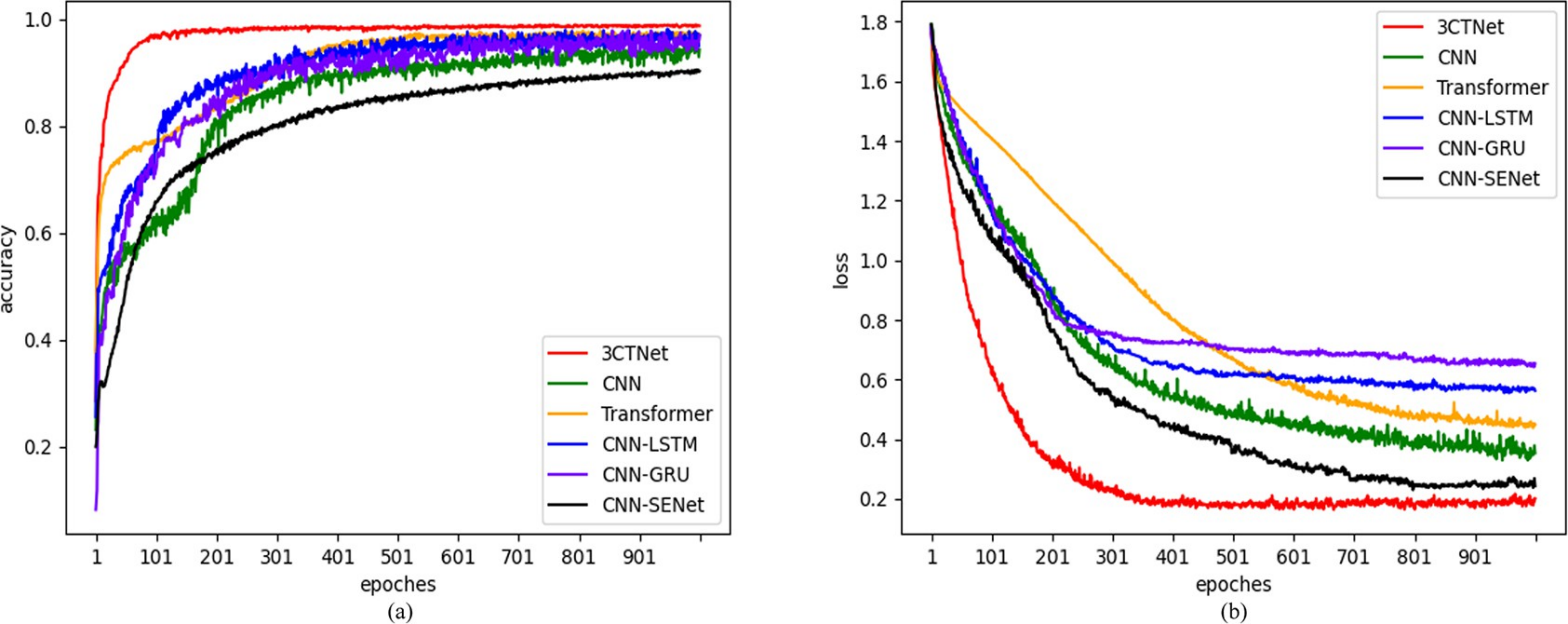
Advanced methods for comparing accuracy curves and loss curves. (a) Accuracy of multiple model comparison. (b) Loss of multiple model comparison.

To highlight the recognition advantages of our proposed model, 3CTNet, has been validated through comparison with other models, including pure CNN, Transformer, CNN-SENet, LSTM, and GRU. The comparative accuracy and loss curves for the model are shown in (Fig 12). The results showed that 3CTNet exhibited rapid improvement in recognition accuracy within the first 200 epochs, eventually reaching a high and stable level. Loss curve converged quickly and reached a stable and low loss rate. Then, we used the datasets obtained from each of the five data enhancement methods to verify the performance of the comparison models, and the experimental results are shown in Table 6. The proposed model achieves an accuracy of 98.876% in recognising the data processed by the ADASYN method, compared to 92.596% for CNN, 96.527% for Transformer, 93.859 for LSTM, 93.698 for GRU, and 89.999% for CNN-SENet. Based on these comparative tests, it can be concluded that the proposed model structure, 3CTNet, demonstrates significant recognition advantages over the other models, and the ADASYN data processing method has a good feature enhancement effect.

**Table 6:** Classification accuracies (%) by comparing models using five data enhancement methods.

	Original data	Kmeans	SVM	SMOTE	Ramdom	ADASYN
**CNN**	86.876	90.035	91.835	88.866	91.619	92.596
**Transformer**	83.586	89.834	95.194	94.846	96.825	96.527
**LSTM**	89.329	95.977	95.361	91.615	91.863	93.859
**GRU**	90.986	91.803	93.104	92.033	93.208	93.698
**CNN-SENet**	82.973	86.265	87.111	86.487	89.173	89.999
**3CTNet**	90.053	94.257	96.901	96.203	97.652	98.876

**Fig 13:**
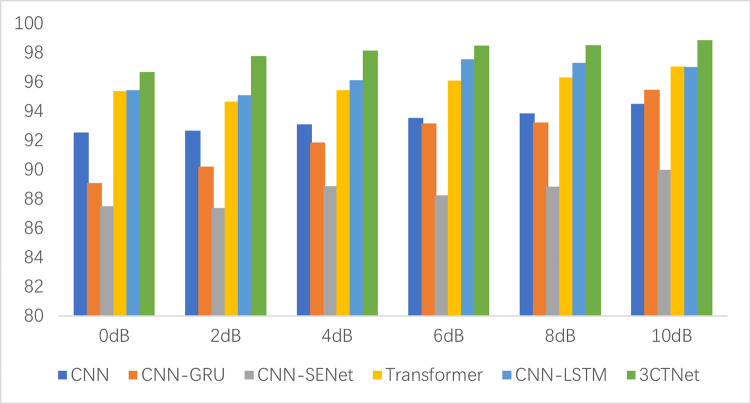
The accuracy of models under different noise interferences.

The recognition accuracy of the validated model under noise interference with different signal-to-noise ratios (SNR) is illustrated in (Fig 13), where the noise gradually decreases and the signal quality gradually increases as the SNR rises. Compared with other models, 3CTNet still shows better recognition accuracy under high noise interference. Therefore, in a strong noise environment, the deep Transformer model 3CTNet can well mitigate the sensitivity to noise, with higher noise resistance and feature learning ability.

## Results and discussion

All results are from a series of experiments carried out in the laboratory with measured AE signals. The sample size of the dataset used here is appropriate for the parameters of the chosen model, and may require further adjustment if models with more complex architectures and larger numbers of parameters are used. Overall, analyzing the above experimental procedure, the rupture process of the rock specimen under Brazilian splitting conditions can be divided into five stages, and the AE energy signals released at each stage with distinctive features can be effectively recognized by the Deep Transformer model and achieve a high recognition accuracy.

The AE signals obtained from the laboratory were processed using wavelet thresholding for noise reduction and oversampling and then used as input data for the deep learning network model. This study emphasizes the significance of the model’s ability to capture long-term dependencies, considering both the historical and future context of the AE process. Understanding the long-term contextual information in the AE signal is crucial, and the stress state of reservoir rock cracks can help determine the direction of crack expansion and guide project construction, thereby improving energy extraction efficiency. It should be noted that the deep learning model for rock type recognition based on AE signals is just a tool, and its application in reservoir research is the primary focus of our research group. Future studies will concentrate on the utilization of AE signals in reservoir evaluation and diagenesis, aiming to develop a set of quantitative and accurate reservoir application methods based on intelligent processing of AE signals.

## Conclusion

This study proposed a new multimodal called 3CTNet for recognizing different AE signals released from six types of rock fracturing experiments. This is the first attempt to apply a variant of the transformer to rock fracturing AE signal recognition. The model establishes relationships within and between modalities based on the five stages of AE signal generation and utilizes oversampled datasets. Experimental results on the comparative dataset demonstrate the effectiveness of 3CTNet. Through a series of experiments and comparative data analysis, the following conclusions can be drawn:

Firstly, experiments were conducted on five data oversampling methods for AE data, and found that high-quality datasets provide a solid foundation for accurate rock type recognition using intelligent models. Among the five oversampling methods, the ADASYN method maximizes the recognition accuracy of the model, with results surpassing the other five methods by 1.8% to 8.8%.

Secondly, a deep transformer model named 3CTNet was innovatively developed, specifically designed to efficiently recognize rock types based on AE signals.The model achieves a recognition accuracy of 98.78% on the oversampled dataset. This innovative application of a deep convolutional transformer model to multimodal AE signal recognition for rock types demonstrates the feasibility and effectiveness of the proposed model architecture.

Furthermore, the ADASYN oversampling method plays a significant role in expanding and balancing the AE signal dataset. The proposed method was the first to identify rock types based on AE signals with a transformer and set a new record. This provides a reference for further research on the embedded recognition system of rock fracturing AE signals in the future. Research based on the embedded system of rock fracturing AE signals will provide convenience for resource explorers’ field operations.

The current study relies on data collected within a controlled laboratory environment. Moving forward, the research will shift its focus towards exploring the diversity of AE data in real-world settings. Future investigations will involve capturing AE signals directly from engineering sites, aiming to enhance the efficiency of rock type identification by incorporating additional parameters. Leveraging the strengths of deep learning algorithms and advancements in computer hardware, the research aims to propel its development towards embedded applications. This approach will facilitate the identification of rock types in offline engineering sites, offering increased convenience and practicality.
